# Effect of Perineural Injection with Different Dextrose Volumes on Median Nerve Size, Elasticity and Mobility in Hands with Carpal Tunnel Syndrome

**DOI:** 10.3390/diagnostics11050849

**Published:** 2021-05-09

**Authors:** Meng-Ting Lin, I-Chun Liu, Wei-Ting Syu, Po-Ling Kuo, Chueh-Hung Wu

**Affiliations:** 1 Department of Physical Medicine and Rehabilitation, National Taiwan University Hospital Hsin-Chu Branch, Hsinchu 300, Taiwan; b96401093@ntu.edu.tw; 2 Department of Physical Medicine and Rehabilitation, National Taiwan University Hospital, College of Medicine, National Taiwan University, Taipei 100, Taiwan; jacy50521@gmail.com (I.-C.L.); polingkuo@gmail.com (P.-L.K.); 3 Graduate Institute of Biomedical Electronics and Bioinformatics, National Taiwan University, Taipei 100, Taiwan; r06945032@ntu.edu.tw; 4 Department of Electrical Engineering, National Taiwan University, Taipei 100, Taiwan

**Keywords:** median nerve, nerve compression syndromes, elasticity imaging techniques

## Abstract

This study aimed to investigate the effect of different injectate volumes on ultrasonographic parameters and the correlation to clinical outcomes under perineural dextrose injection (PDI). In this post hoc analysis of the randomized, double-blinded, three-arm trial, ultrasound-guided PDI with either 1 mL, 2 mL, and 4 mL 5% dextrose water was administered, respectively, in 14, 14, and 17 patients. Ultrasound outcomes included mobility, shear-wave elastography (SWE), and cross-sectional area (CSA) of the median nerve; clinical outcomes were Visual Analog Scale (VAS) and Boston Carpal Tunnel Questionnaire (BCTQ) score. Outcomes were measured before injection, and after injection at the 1st, 4th, 12th, and 24th week. For ultrasound outcomes, CSA decreased significantly from baseline data at all follow-up time-points in the 2 mL group (*p* = 0.005) and the 4 mL group (*p* = 0.015). The mean change of mobility from baseline showed a greater improvement on the 4 mL group than the other groups at the 1st week post-injection. For clinical outcomes, negative correlation between the VAS and mobility at the 1st (*p* = 0.046) and 4th week (*p* = 0.031) post-injection in the 4 mL group were observed. In conclusion, PDI with higher volume yielded better nerve mobility and decreased CSA of median nerve, but no changes of nerve elasticity.

## 1. Introduction

Carpal tunnel syndrome (CTS) occur with the symptoms of intermittent nocturnal paresthesia and dysesthesia, followed by loss of sensation, weakness, and thenar muscle atrophy in later stages [1]. Although etiology of CTS is multifactorial, compression of the median nerve in space-limited osteofibrous canal at wrist level was proposed [1,2]. The gold standard of diagnosis is electrophysiological study, but the ultrasonography was applied in recent decades for better approach to morphology change of median nerve. The cross-sectional area of median nerve became another diagnostic option for CTS [3]. In addition, dynamic ultrasound was also a useful tool to assess the flexor tendon and median nerve motion in response to finger and hand movement [4,5]. Previous review demonstrated reducing excursion of median nerve in CTS patients [6]. Furthermore, the elasticity of nerve was also an important parameter to evaluate entrapment neuropathies. A systemic review revealed the median nerve became stiffer after compression under shear wave elastography quantification [7,8]. Shear wave elastography had high sensitivity to detect changes in median nerve elasticity [9].

Non-operative treatments such as wrist splint, medications, and steroid injection would be initially administered in most patients with mild to moderate symptoms and disability. Recent studies demonstrated the perineural dextrose injection (PDI), compared to steroid, improved pain and disability significantly for the CTS group [10]. A higher volume of PDI yielded better clinical outcomes in our previous work [11]. Moreover, injection therapy with hydrodissection could also improve nerve displacement after decreasing nerve gliding resistance in the carpal tunnel [12]. However, the dynamic ultrasonographic change and elastography of median nerve after a different volume of dextrose injection is still unknown. Thus, we aimed to investigate the effect of different injectate volumes on ultrasonographic parameters and the correlation to clinical outcomes under PDI in patient with carpal tunnel syndrome. We hypothesized larger volume might improve ultrasonographic parameters of CTS, which could be predicted by clinical outcomes.

## 2. Materials and Methods

### 2.1. Study Design

We performed this post hoc analysis of previous double-blinded three-arm randomized control trial [11] for further ultrasonographic outcomes investigation. The study was approved by the institutional review board of National Taiwan University Hospital on July 26, 2018, and registered on ClinicalTrials.gov (registration number: NCT03598322). We conducted the research in accordance with the Declaration of Helsinki and received informed consent from each participant in our study. We enrolled patients from June 2018 to December 2019. Diabetic patients were excluded for post hoc analysis.

### 2.2. Blinding

The patients, outcome assessors, and research assistants were all blinded to treatment allocation, while the physician performing injection was the only one aware of the treatment allocations.

### 2.3. Inclusion and Exclusion Criteria

Inclusion criteria were (1) aged 20–80 years, (2) diagnosed with idiopathic CTS, and (3) fulfilled the electrophysiological criteria and clinical criteria [11]. Exclusion criteria were patients with diabetes mellitus, previous wrist surgery, traumatic wrist injury within 2 years, previous wrist injection within 3 months, history of peripheral nerve injuries (brachial plexopathy, cervical radiculopathy or thoracic outlet syndrome), history of autoimmune disease, and inability to cooperate with study protocol.

### 2.4. Randomization

We performed randomization in permuted blocks of six. The allocation process was sealed by the independent research assistant. The participants were assigned to either 1 mL group, 2 mL group, or 4 mL group where they received a corresponding volume of ultrasound-guided PDI. After the injection, patients were allowed to take paracetamol, but non-steroidal anti-inflammatory drugs or neuropathic pain medications were prohibited. We did not provide additional physiotherapy, occupational therapy or night splint.

### 2.5. Ultrasound-Guided PDI

The corresponding author, a physiatrist with experience of more than 8000 cases of ultrasound-guided intervention, performed all ultrasound-guided PDIs with Aplio 500 ultrasound system (APLIO 500 TUS-A500, Toshiba Medical Systems Corporation, Tochigi, Japan). All participants were positioned under the supinated forearm position. The median nerve was identified at the proximal inlet of carpal tunnel. After aseptic preparation, the 25-gauge needle was inserted from the radial side with an in-plane approach. Half volume of the 5% dextrose water (D5W) was injected to separate the median nerve and flexor retinaculum and the other half below the median nerve [11]. The total D5W injected was either 1 mL, 2 mL, or 4 mL with the covered syringe to achieve a better blinding process.

### 2.6. Outcome Assessment

We collected patients’ demographics, medical history, symptom duration, clinical outcomes (Visual Analog Scale (VAS) and Boston Carpal Tunnel Questionnaire (BCTQ) score) and ultrasound outcomes (mobility, shear-wave elastography (SWE) and cross-sectional area (CSA) of the median nerve) before injection, and after injection at the 1st, 4th, 12th, and 24th week.

#### 2.6.1. Ultrasound Outcome

**Figure 1:**
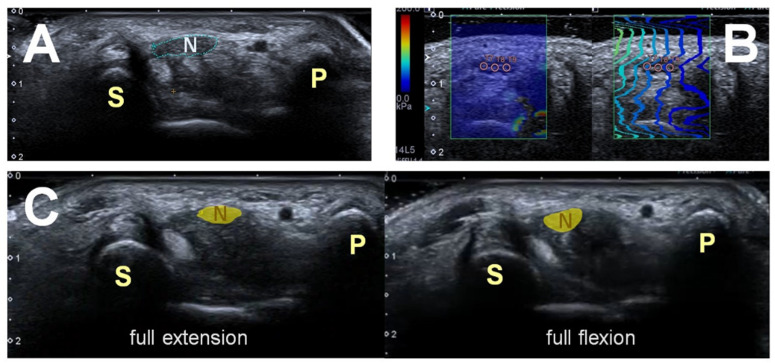
Measurement of ultrasound outcomes with (**A**) cross-section area (CSA), (**B**) shear-wave elastography (SWE), and (**C**) centroid position of the median nerve. (**A**) CSA of median nerve is measured at the carpal tunnel inlet level between scaphoid bone (S) and pisiform bone (P). (**B**) SWE of median nerve is measured at the carpal tunnel inlet level. Red color implies stiffer tissue and blue color implies soft tissue. Circular region is for quantitation of tissue stiffness in kilopascals (kPa) units. (**C**) Distance of the centroid position of the median nerve between the finger extension and full flexion position is recorded on ultrasound video. P = Pisiform bone. S = Scaphoid bone. N = Median nerve.

Ultrasound examinations were performed by the first author, a physiatrist with more than 4 years ultrasound experience with an Aplio 500 ultrasound system. Patients were instructed to keep a supinated forearm, fully extended fingers, neutral wrist, and shoulder position. All images were captured under transverse view of the median nerve at the carpal tunnel inlet level (between the pisiform and scaphoid bones) with 7–14 MHz linear array transducer (Figure 1). The transducer was perpendicular to median nerve and maintained slight contact with skin to minimize carpal tunnel compression artifacts.

CSA of Median Nerve 

Ultrasound measurements of the median nerve CSA at the carpal tunnel inlet level was performed. The CSA was calculated by the ultrasound machine after using a caliper to encompass the median nerve manually (Figure 1A).

SWE of Median Nerve 

The assessor placed the transducer perpendicular to the wrist without pressure and waited until acquiring stable image quality. SWE was performed under the patient′s initial position. In the SWE mode, the machine showed a real-time color elasticity map to detect the tissue stiffness, where stiffer tissue was indicated by red and softer tissue by blue color. For the quantitation of tissue stiffness, circular region of interest was used to obtain mean shear modulus expressed in kilopascals (kPa) units. Data was excluded if areas with large signal loss. We collected every wrist for three times to drive average value (Figure 1B).

Mobility of Median Nerve

The participants were placed in the aforementioned position initially. They were instructed to make a fist with full flexion while keeping a neutral wrist, and then back to initial finger extension position. Each trial was finished in 4 s and the patient was asked to maintain a constant speed. Six cycles of movement were collected. The transverse mobility of the median nerve in the carpal tunnel was measured according to methods described in previous studies [13,14,15]. Distance of the centroid position of the median nerve between the initial and next frames of the ultrasound video was digitally quantified with the interval of thirty frames per second. Sequential displacements between successive frames were calculated to represent the excursion of the median nerve (Figure 1C).

#### 2.6.2. Clinical Outcome: VAS and BCTQ Score

The continuous VAS was applied to evaluate pain or paresthesia severity with a score of 10 means unbearable and a score of 0 means no pain/paresthesia at all. We adopted the maximal pain VAS as score measurement. BCTQ Score includes 11 questions symptom severity scale and eight questions functional status scale. Each question was answered on a scale of 1 to 5, with larger numbers representing more severe and unable to perform certain activities. We utilized the sum of all the questions as the outcome indicator.

### 2.7. Intra-Rater and Inter-Rater Reliability

We utilized the aforementioned preparation protocol to examine five non-enrolled healthy individuals to measure nerve CSA, mobility, and elastography. Healthy individuals were evaluated by the first author and another examiner (with a 4-year experience of musculoskeletal ultrasound) on the same day, and reevaluated by the first author on the second day. We analyzed the data to compute the intraclass correlation coefficient ((ICC)2,1) of CSA, mobility, and elastography, respectively. The intra-rater and inter-rater reliability were determined and classified as poor (ICC, 0.00–0.20), fair (ICC, 0.20–0.40), good (ICC, 0.40–0.75), and excellent (ICC > 0.75) [16]. We calculated minimal detectable change (MDC) at a 95% confidence interval (CI) with the formula: MDC_95_ = 1.96 × $\sqrt{2}$× standard error of measurement (SEM), where SEM = standard deviation(SD) pooled ×$\;\sqrt{1-ICC}$) [17].

### 2.8. Statistical Analyses

All data analyses were performed with SPSS Statistics v.22.0. We calculated sample size with G*power 3.1.9.4 for preliminary power analysis and at least 14 wrists in each group were required to achieve sufficient power. Preliminary Shapiro–Wilks test revealed normal distributed samples, and Levene′s Test for homogeneity of variance was performed to determine if equal variances. One-way ANOVA was used for between-group analysis and one-way repeated-measures ANOVA for within-group analysis in continuous data, with the Tukey test for post hoc analysis. Chi-square test for categorical data, repeated measures ANOVA for sequential data. All statistical tests were 2-tailed, and a *p* value of less than 0.05 was considered statistically significant.

## 3. Results

### 3.1. Clinical Characteristics

Among the 33 participants with 51 wrists that were assessed for eligibility into the trial, four participants were excluded due to rejection to injection. Therefore, a total of 45 wrists were randomized into three groups (Figure 1). All participants received a completed follow-up of up to 24 weeks after injection. There was no significant difference between the three groups in age, gender, hypertension, symptom duration, ultrasound outcomes or clinical outcomes (Table 1).

### 3.2. Intra-Rater, Inter-Rater Reliability and MDC

The intra-rater reliability of the CSA (mm^2^), mobility (mm), and elastography (kPa) measurements were all excellent (ICC of CSA = 0.895 (95% CI: 0.568–0.988); ICC of mobility = 0.818 (95% CI: 0.367–0.978); ICC of elastography = 0.851 (95% CI: 0.667–0.979)). The interrater reliability of the CSA, mobility, and elastography measurements were good to excellent (ICC of CSA = 0.936 (95% CI: 0.831–0.992); ICC of mobility = 0.602 (95% CI: 0.014–0.946); ICC of elastography = 0.480 (95% CI: 0.041–0.916)). The MDC_95_ of the CSA, mobility, and elastography were 3.276 (mm2), 0.144 (mm), and 28.51(kPa), respectively.

### 3.3. Within-Group PDI Effects

For ultrasound outcomes, CSA decreased significantly from baseline data at all follow-up time-points in the 2 mL group (*p* = 0.005) and the 4 mL group (*p* = 0.015) (Table 2); however, the decrease of CSA in both groups did not reach the MDC_95_ of CSA measurement. No statistical difference was observed for mobility or elastography; but after the post hoc analysis, it showed that mobility significantly increased at the 1st (mean (SD): 1.17 (0.77), *p* = 0.023), 4th (mean(SD): 1.12 (0.81), *p* = 0.006), and 12th (mean(SD): 1.12 (0.88), *p* = 0.033) weeks post-injection from baseline (mean(SD): 0.81 (0.54)) in the 4 mL group (Table 2); the elastography significantly decreased at the 24th (mean(SD): 33.60 (16.52), *p* = 0.042) weeks post-injection from baseline (mean(SD): 51.29 (35.70)) in the 4 mL group (Table 2). For clinical outcomes, VAS and BCTQ significantly improved at all time-points in three groups (Table 3).

### 3.4. Between-Group PDI Effects

In the 4 mL group, the mean change of mobility from baseline showed a greater improvement than the other groups at the 1st week post-injection (4 mL: 0.35 (0.58), 2 mL: -0.12 (0.56), 1 mL: 0.01 (0.39)), where it reached the MDC_95_ value and represented clinical significance; in addition, the mean change of VAS and BCTQ from baseline improved more compared to other groups at the 1st and 4th week post-injection (Table 4).

### 3.5. Correlation between Ultrasound and Clinical Outcomes

The correlation analysis showed statistically significant negative correlation between the VAS and mobility of the median nerve at the 1st (correlation coefficient R = −0.490, *p* = 0.046) and 4th week (correlation coefficient R = −0.522, *p* = 0.031) post-injection in the 4 mL group. No relationship between the other ultrasound and clinical outcomes was observed.

## 4. Discussion

Our study revealed CSA of median nerve decreased significantly at all follow-up time-points in the 2 mL group and the 4 mL group. The mean change of mobility from baseline showed a greater improvement on the 4 mL group at the 1st week post-injection. For clinical outcomes, VAS and BCTQ significantly improved at all time-points in three groups. Negative correlation between the VAS and mobility was noticed at the 1st and 4th week post-injection in the 4 mL group.

We reported significantly increased mean change of nerve mobility from baseline at the 1st weeks post-injection in the 4 mL group. Systematic review evidenced the reduced mobility of median nerve in people with CTS compared to healthy controls [18]. Hence, treatments such as hydrodissection to directly influence median nerve excursion may play an important role. Hydrodissection enhanced nerve excursion through mechanical separation of subsynovial connective tissue(SSCT) [19]. In aspect of mechanical concept, cadaveric study assessing the biomechanical change founded the peak gliding resistance reduced after median nerve hydrodissection [12]. The theory based on nerve separation from surrounding connective tissue to release entrapment by perineural injection [20]. Evidence implied larger injection volume had greater distribution and higher pressure to disrupt adhesion and fibrosis of subsynovial connective tissue; it provided higher potential to reduce the rate of treatment failure within 1 year [21]. A serial of studies demonstrated good treatment response of PDI for CTS with either 5 mL or 10 mL D5W [10,22]. Even hydrodissection with solitary normal saline could deploy therapeutic effect for the pre-surgical stage [23]. Therefore, our research demonstrated higher volume of PDI increased nerve movement by means of hydrodissection. 

In our study, there was no significant difference on elastography at all time points after any volume of PDI. Systematic review concluded CTS patients presented stiffer median nerves at the wrist level than healthy subjects [7]. Previous study revealed no significant change in the median nerve stiffness after steroid injection, but decreased stiffness in whole carpal tunnel contents [24]. Another research reported similar results with steroid injection, where the median nerve remained stiff, but with softer intracarpal tunnel contents surrounding the nerve [25]. To explain the unchanged nerve stiffness after treatment, irreversible internal fibrosis of nerve sheaths and axons after prolonged nerve compression was assumed [26]. Furthermore, steroid injection surrounding the nerve could soften intracarpal tunnel contents by decreasing the collagen, fibroblast proliferation, and promotion of glycosaminoglycan synthesis [27] Our study was the first one to investigate the effect of dextrose injection on median nerve elasticity and exhibited limited change of nerve stiffness after PDI.

A significant negative correlation was observed between the VAS and mobility of the median nerve at the 1st and 4th week post-injection in the 4 mL group. Schrier et al. showed the association between median nerve mobility and symptom severity at 3 months post-surgery [5]. Another study found significant negative correlation between the transverse sliding distance of median nerve and pain score after steroid intervention [28]. These results allowed us to monitor therapeutic effect not only with subjective pain score, but also nerve mobility to provide further morphological evidence after injection. Our study revealed there was no correlation between BCTQ and ultrasound parameters. Similarly, previous research showed that the functional scale was not correlated to diameter, circumference, or CSA in CTS patients, implying that not only recovery of nerve characteristics, but also physiological restoration (muscle endurance, strength, intra-tunnel pressure, and bone alignment of wrist) contributed to functional improvement [29]. On the other hand, despite a significant correlation between VAS and mobility, symptom severity subscale (SSS) of BCTQ was not correlated to ultrasound measurements in our research. SSS of BCTQ contained 11 items; 1st–5th items were pain-related questions and 6th–10th items were paresthesia‑relevant symptoms. As a result, the VAS could only be used to evaluate the severity of pain, corresponding to the 1st–5th items of SSS, instead of covering overall SSS.

There are several clinical implications. First, D5W showed notable effectiveness on clinical outcomes including VAS and BCTQ. Second, PDI with higher volume could provide brief improvement on mobility of median nerve. Third, owing to negative correlation between nerve mobility and VAS, the ultrasonographic parameter might be applied to predict the severity of clinical manifestation. 

There were several limitations in this study. First, to standardize our measurement on nerve displacement, participants were instructed to make a fist with full flexion, then extend a finger to initial position. However, either the patient’s mid forearm or hand was not constrained. Therefore, unnecessary hand movement might appear to influence overall nerve displacement. Patients were instructed to maintain a constant speed during finger flexion. Although there was no extra movement or different flexion speed under our inspection, some undetectable tiny movement was still inevitable. Secondly, the motion of nerve was in three dimensions instead of linear movement. However, our study only focused on transverse mobility rather than longitudinal mobility. Therefore, the overall displacement of the median nerve might be underestimated. Third, the sample sizes of our groups were relatively small. More patients were needed in the future. Fourth, we enrolled some participants with bilateral hands, where one hand might interfere on the subjective outcomes of the other hand.

## 5. Conclusions

In conclusion, PDI with higher volume yielded better nerve mobility and decreased CSA of median nerve, but no changes of nerve elasticity.

## Figures and Tables

**Table 1:** Baseline characteristics between three groups.

	4 mL Group (*n* = 17)	2 mL Group (*n* = 14)	1 mL Group (*n* = 14)	*p*-Value ^†^
Age (SD)	56.9 (9.1)	52.9 (10.1)	59.2 (8.1)	0.195
Female (%)	94.1	85.7	85.7	0.281
Hypertension (%)	23.5	21.4	42.9	0.352
Symptom duration (SD)	66.0 (77.5)	21.9 (31.4)	58.4 (60.9)	0.144
**Ultrasound outcomes**				
CSA (SD), mm^2^	14.1 (2.8)	14.7 (3.6)	14.4 (4.0)	0.885
Elastography (SD), kPa	51.3 (35.7)	50.6 (29.3)	63.0 (40.1)	0.577
Mobility (SD), mm	0.8 (0.5)	1.2 (0.6)	0.9 (0.4)	0.127
**Clinical outcomes**				
VAS (SD)	5.6 (1.5)	6.2 (1.6)	5.2 (1.9)	0.259
BCTQ (SD)	46.3 (9.1)	43.4 (14.3)	38.5 (14.0)	0.252

^†^ Between-group comparison: one- way ANOVA for continuous data and chi-square test for categorical data. Abbreviation: SD, standard deviation; VAS, visual analog scale; BCTQ, Boston Carpal Tunnel Syndrome Questionnaire; CSA, cross-sectional area of median nerve.

**Table 2:** Ultrasound outcome in the three groups.

Outcomes	Baseline	1W	4W	12W	24W	*p*-Value ^†^
**CSA (mm^2^)**						
1 mL group	14.23 (4.19)	13.92 (4.17)	13.61 (2.26)	12.08 (2.66)	12.77 (3.75)	0.080
2 mL group	14.68 (3.56)	14.00 (3.33)	13.43 (2.53)	13.14 2.32)	12.07 (2.16)	0.005 **
4 mL group	13.88 (2.80)	12.75 (2.44)	12.81 (3.15)	13.06 (3.00)	11.63 (2.06)	0.015 *
**Elasticity (kPa)**						
1 mL group	62.97 (40.07)	49.43 (31.94)	49.34 (30.45)	47.99 (26.23)	56.79 (39.03)	0.651
2 mL group	50.58 (29.34)	42.00 (24.08)	48.15 (26.27)	50.25 (29.58)	50.03 (23.85)	0.859
4 mL group	51.29 (35.70)	43.76 (28.68)	55.14 (28.9)	50.48 (36.36)	33.60 (16.52)	0.081
**Mobility (mm)**						
1 mL group	0.92 (0.42)	0.92 (0.55)	0.95 (0.43)	1.02 (0.52)	1.06 (0.79)	0.873
2 mL group	1.21 (0.63)	1.09 (0.42)	1.17 (0.69)	1.05 (0.49)	1.03 (0.58)	0.487
4 mL group	0.81 (0.54)	1.17 (0.77)	1.12 (0.81)	1.12 (0.88)	1.10 (0.94)	0.188

^†^ One-way repeated-measures ANOVA was used for within-group analysis. The data was presented as mean (standard deviation). Abbreviation: CSA, cross-sectional area of median nerve. * *p* < 0.05, ** *p* < 0.01.

**Table 3:** Clinical outcomes in three groups: mean VAS and BCTQ.

	Baseline	1W	4W	12W	24W	*p*-Value ^†^
**VAS**						
1 mL group	5.21 (1.86)	3.25 (1.98)	3.82 (1.81)	3.21 (1.50)	3.43 (2.42)	0.003 *
2 mL group	6.25 (1.57)	4.66 (2.00)	4.07 (2.58)	3.11 (2.08)	2.57 (1.91)	<0.001 *
4 mL group	5.62 (1.55)	2.91 (1.94)	1.91 (1.86)	1.79 (2.04)	2.65 (2.42)	<0.001 *
**BCTQ**						
1 mL group	39.43 (13.96)	29.64 (10.29)	29.79 (12.14)	27.29 (7.54)	27.64 (7.95)	<0.001 *
2 mL group	43.36 (14.26)	33.93(11.68)	29.36 (11.11)	27.14 (11.23)	25.50 (9.73)	<0.001 *
4 mL group	46.65 (8.87)	29.47 (8.95)	23.41 (4.58)	23.88 (4.97)	29.59 (11.84)	<0.001 *

^†^ One-way repeated-measures ANOVA was used for within-group analysis. The data was presented as mean (SD). Abbreviation: SD, standard deviation; VAS, visual analog scale; BCTQ, Boston Carpal Tunnel Syndrome Questionnaire. * *p* < 0.01.

**Table 4:** Comparison of mean change from baseline in outcomes between three groups.

	4 mL Group (*n* = 17)	2 mL Group (*n* = 14)	1 mL Group (*n* = 14)	*p*-Value ^†^
**Ultrasound outcomes**				
**Δ CSA (mm^2^)**				
1 weeks	−1.21 (1.97)	−0.68 (2.32)	−0.31 (1.60)	0.499
4 weeks	−0.79 (3.12)	−1.50 (2.93)	−0.62 (3.31)	0.746
12 weeks	−1.31 (2.50)	−1.54 (3.19)	−2.15 (3.02)	0.748
24 weeks	−2.14 (2.44)	−2.32 (3.36)	−0.77 (1.88)	0.267
**Δ Elastography (kPa)**				
1 weeks	−7.53 (26.35)	−8.58 (31.69)	−13.53 (38.10)	0.863
4 weeks	−3.85 (23.36)	−2.43 (33.62)	−13.63 (40.52)	0.338
12 weeks	−0.81 (45.93)	−0.33 (40.71)	−14.98 (46.82)	0.610
24 weeks	−17.69 (34.79)	−0.55 (34.30)	−6.18 (43.25)	0.434
**Δ Mobility (mm)**				
1 weeks	0.35 (0.58)	−0.12 (0.56)	0.01 (0.39)	0.040 *
4 weeks	0.31 (0.40)	−0.04 (0.47)	0.04 (0.39)	0.060
12 weeks	0.31 (0.54)	−0.16 (0.50)	0.11 (0.52)	0.058
24 weeks	0.28 (0.64)	−0.18 (0.49)	0.14 (0.55)	0.084
**Clinical outcomes**				
**Δ VAS (SD)**				
1 weeks	−2.71 (1.77)	−1.59 (1.65)	−1.96 (1.68)	0.188
4 weeks	−3.71 (2.19)	−2.18 (2.85)	−1.39 (1.68)	0.023 *
12 weeks	−3.82 (1.78)	−3.14 (3.00)	−2.00 (1.59)	0.079
24 weeks	−2.97 (2.37)	−3.68 (2.37)	−1.79 (2.28)	0.108
**Δ BCTQ (SD)**				
1 weeks	−17.18 (8.67)	−9.43 (12.52)	−9.79 (7.05)	0.048 *
4 weeks	−23.24 (8.14)	−14.00 (14.75)	−9.64 (6.97)	0.002 **
12 weeks	−22.77 (9.40)	−16.21 (15.92)	−12.14 (10.20)	0.055
24 weeks	−17.06 (12.95)	−17.86 (13.06)	−1.79 (12.43)	0.397

^†^ Between-group comparison: one-way ANOVA was used for statistical analysis. Abbreviation: SD, standard deviation; VAS, visual analog scale; BCTQ, Boston Carpal Tunnel Syndrome Questionnaire; CSA, cross-sectional area of median nerve. * *p* < 0.05, ** *p* < 0.01.

## Data Availability

Data Availability Statements in section “MDPI Research Data Policies” at https://www.mdpi.com/ethics.
